# Carbidopa, a drug in use for management of Parkinson disease inhibits T cell activation and autoimmunity

**DOI:** 10.1371/journal.pone.0183484

**Published:** 2017-09-12

**Authors:** Huabin Zhu, Henrique Lemos, Brinda Bhatt, Bianca N. Islam, Abhijit Singh, Ashish Gurav, Lei Huang, Darren D. Browning, Andrew Mellor, Sadanand Fulzele, Nagendra Singh

**Affiliations:** 1 Department of Biochemistry and Molecular Biology, Medical College of Georgia, Augusta University, Augusta, Georgia, United States of America; 2 Institute of Cellular Medicine, Newcastle University, Newcastle-upon-Tyne, United Kingdom; 3 Department of Emergency Medicine, Medical College of Georgia, Augusta University, Augusta, Georgia, United States of America; 4 University of Pennsylvania School of Dental Medicine, Philadelphia, PA, United States of America; 5 Department of Orthopedics Surgery, Medical College of Georgia, Augusta University, Augusta, Georgia, United States of America; Wayne State University, UNITED STATES

## Abstract

Carbidopa is a drug that blocks conversion of levodopa to dopamine outside of central nervous system (CNS) and thus inhibits unwanted side effects of levodopa on organs located outside of CNS during management of Parkinson’s Disease (PD). PD is associated with increased expression of inflammatory genes in peripheral and central nervous system (CNS), infiltration of immune cells into brain, and increased numbers of activated/memory T cells. Animal models of PD have shown a critical role of T cells in inducing pathology in CNS. However, the effect of carbidopa on T cell responses *in vivo* is unknown. In this report, we show that carbidopa strongly inhibited T cell activation *in vitro* and *in vivo*. Accordingly, carbidopa mitigated myelin oligodendrocyte glycoprotein peptide fragment 35–55 (MOG-35-55) induced experimental autoimmune encephalitis (EAE) and collagen induced arthritis in animal models. The data presented here suggest that in addition to blocking peripheral conversion of levodopa, carbidopa may inhibit T cell responses in PD individuals and implicate a potential therapeutic use of carbidopa in suppression of T cell mediated pathologies.

## Introduction

Parkinson’s disease (PD) is characterized by a loss of dopaminergic neurons in substantia nigra in the brain, resulting in decreased production of the neurotransmitter and messenger dopamine. Loss of dopamine is the central to the development of PD [1–4]. Levodopa, the dopamine precursor, is converted into dopamine by L-dopa decarboxylase (DDC). This leads to increased production of dopamine. Therefore, levodopa is very effective in the management of Parkinson’s disease [3–6]. DDC is expressed by neurons in the central nervous system, liver, kidney, pancreas, and T lymphocytes [7–9]. Consumption of levodopa results in systemic production of dopamine, which limits production of dopamine in the central nervous system. In addition, activation of peripheral dopamine receptors results in nausea and vomiting. Carbidopa is an inhibitor of DDC and does not cross the blood-brain barrier, thus preferentially inhibiting the conversion of levodopa to dopamine outside of the brain. However, the effects of carbidopa on peripheral cells, specifically T lymphocytes, have not been studied in detail.

Under steady state conditions DDC catalyzes two reactions: 1) metabolism of tyrosine by DDC is a key step in production of catecholamines; dopamine, epinephrine and norepinephrine, and 2) DDC also catalyzes conversion of 5-hydroxytryptophan to serotonin. T lymphocytes express DDC mRNA and functional enzyme and produce both dopamine and serotonin [10–13]. Naïve T cells express type 7 5-hydroxytryptamine receptor (5-HT_7_R), whereas activated T cells express 5-HT_7_R, 5-HT_1B_R and 5-HT_2A_R, which upon activation by serotonin transduces signal to T cells [11]. Serotonin enhances the activation of T cells [11]. T cell express both D1 and D2 class of dopamine receptors [14]. Acting through D1 receptors, dopamine inhibits production of IL-10 and TGF-ß1 by T regulatory cells (Treg cells) leading to enhanced proliferation of conventional T cells [15]. It also induces differentiation of naïve T cells into Th2 lineage [16]. Treatment of naïve T cells with dopamine results in increased adhesion to fibronectin, production of TNF-α, and IL-10 [17]. On the other hand both dopamine and L-dihydroxyphenylalanine (L-dopa), a precursor for dopamine, block mitogen driven proliferation of T cells *in vitro* in a dose dependent manner [10]. High concentration of dopamine inhibits anti-CD3 and IL-3 induced proliferation of human T cells in vitro [18, 19]. These *in vitro* studies demonstrate that DDC activation may either promote or suppress T cells response.

In this study, the effect of carbidopa on T cell responses and subsequent pathology *in vivo* was evaluated. Our data demonstrate that carbidopa blocked T cell responses and suppressed T cell mediated autoimmunity *in vivo*. These finding suggest that carbidopa has a therapeutic potential for alleviation of T cell driven pathologies *in vivo*.

## Material and methods

### Mice

C57BL/6J and DBA/1 mice were obtained from Jackson laboratory and Taconic respectively. The Institutional Animal Care and Use Committee (IACUC), Augusta University approved all animal procedures.

### Experimental autoimmune encephalitis (EAE)

EAE was induced as described previously [20]. In brief, animals were immunized subcutaneously on rear flank (2 sites) with 100 μg of MOG_35–55_/mouse (MEVGWYRSPFSRVVHLYRNGK) (Bio Basic, Canada) emulsified in CFA (BD Biosciences) containing 4 mg/ml *Mycobacterium tuberculosis* H37Ra (BD Biosciences). Animals were injected with pertussis toxin (intraperitoneally, on days 0 and 2 after immunization (200 ng/mouse). All the mice were monitored daily for paralysis, behavior and ability to move, eat or drink. Cages were supplemented with bottled water and petri dish containing food was placed at the floor of cage to facilitate the food and water intake by mice undergoing EAE.

Clinical symptoms of EAE was scored as follows; 0, no clinical symptom; 0.5, partial paralysis of tail; 1, paralysis of tail or wobbling gait; 1.5, partial paralysis of one lower leg and paralysis of tail; 2, paralysis of one lower leg or partial paralysis of both lower leg and paralysis of tail; 2.5, paralysis of one lower leg and partial paralysis other leg and paralysis of tail; 3, paralysis both lower leg and paralysis of tail; 3.5, paralysis of both lower leg, weakness of the upper leg and paralysis of tail; 4, paralysis of 3 legs and tail; 4.5, paralysis of 3 legs, weakness in 4^th^ leg and paralysis of tail; 5, Paralysis of tail, and all four legs /moribund or dead. No animals died prior to the experimental endpoint. Where indicated, mice were given drinking water containing 1.5 mg/ml carbidopa (TCI, Tokyo, Japan).

### Collagen induced arthritis (CIA)

Mice were immunized intradermically at base of tail with bovine collagen type II (Kind gift from Dr. David Brand, University of Tennessee Health Science Center, Memphis, Tennessee) emulsified in complete Freund’s adjuvant (100 μg/mouse). Animals were monitored daily for ability to move, weight loss, erythema and swelling of tarsals, ankle and leg joints and/or ankylosis of the limb. Arthritis was graded for each limb as follows; 0 = no swelling, 1 = mild swelling with erythema, 2 = moderate joint swelling, 3 = severe swelling and digit deformity, and 4 = maximal swelling with ankyloses. Endpoint criteria included severe joint swelling and ankylosis detected on flexion, severely impaired movement, inability to eat and/or drink. No animals died prior to the experimental endpoint. Decalcified bone sections were stained with hematoxylin and eosin for evaluation of joint inflammation or TRAP kit (Sigma 386A-1KT) for osteoclasts.

### Lymphocytes activation assays

Lymph node cells (2 x 10^5^ cells/well) from immunized mice were cultured with indicated amount of antigen in 96 well flat-bottomed plates (Corning, Tewksbury) in 0.2 ml of RPMI fortified with 10% fetal bovine serum (GE Healthcare, Logan, Utah), 10 mM HEPES pH 7.4 (Sigma, St. Louis, MO) and 50μM of 2-mercaptoethanol (Thermo-Fisher, Waltham, MA). At indicated time point, plates were pulsed with 0.5 μci of ^3^H-Thymidine (Perkin Elmer, Waltham, MA) for 6–8 hours and thymidine incorporation was determined. Alternatively, culture supernatant was harvested at indicated time points and presence of indicated cytokines were determined in sandwich. Central nervous system homogenate from MOG_35-55_ immunized mice was overlaid on 40% percoll and spun at 700g for 12 minutes. Cells recovered from pellet were used as immune cells. Immune cells from brain were activated with 100 ng/ml of PMA and 1 μM of ionomycin (EMDmillipore Billerica, MA) in the presence of monensin and brefeldin A (Thermo-Fisher, Waltham, MA). Four hours later cells were stained with antibodies against CD4, IL-17, IFN-γ and analyzed using LSRII flow cytometer (BD Biosciences, San Jose, CA).

### *In vivo* suppression assays

Carboxyfluorescein succinimidyl ester (CFSE, Thermo-Fisher, Waltham, MA)-labeled chicken egg albumin(OVA)-specific (Thy1.1) T cells from OT-2 mice were intravenously injected into C57BL/6 (Thy1.2). One day later, mice were then immunized subcutaneously with OVA (5 **μ**g/mouse) emulsified in CFA. Half of the mice were given carbidopa as above. Three days later, dilution of CFSE was determined on OTII CD4+ T cells using LSR II flow cytometer as above.

### Statistical analysis

Statistical significance was calculated using T-test with two-tailed analysis using Microsoft Excel unless stated otherwise.

## Results

### Carbidopa inhibits T cell proliferation

**Fig 1:**
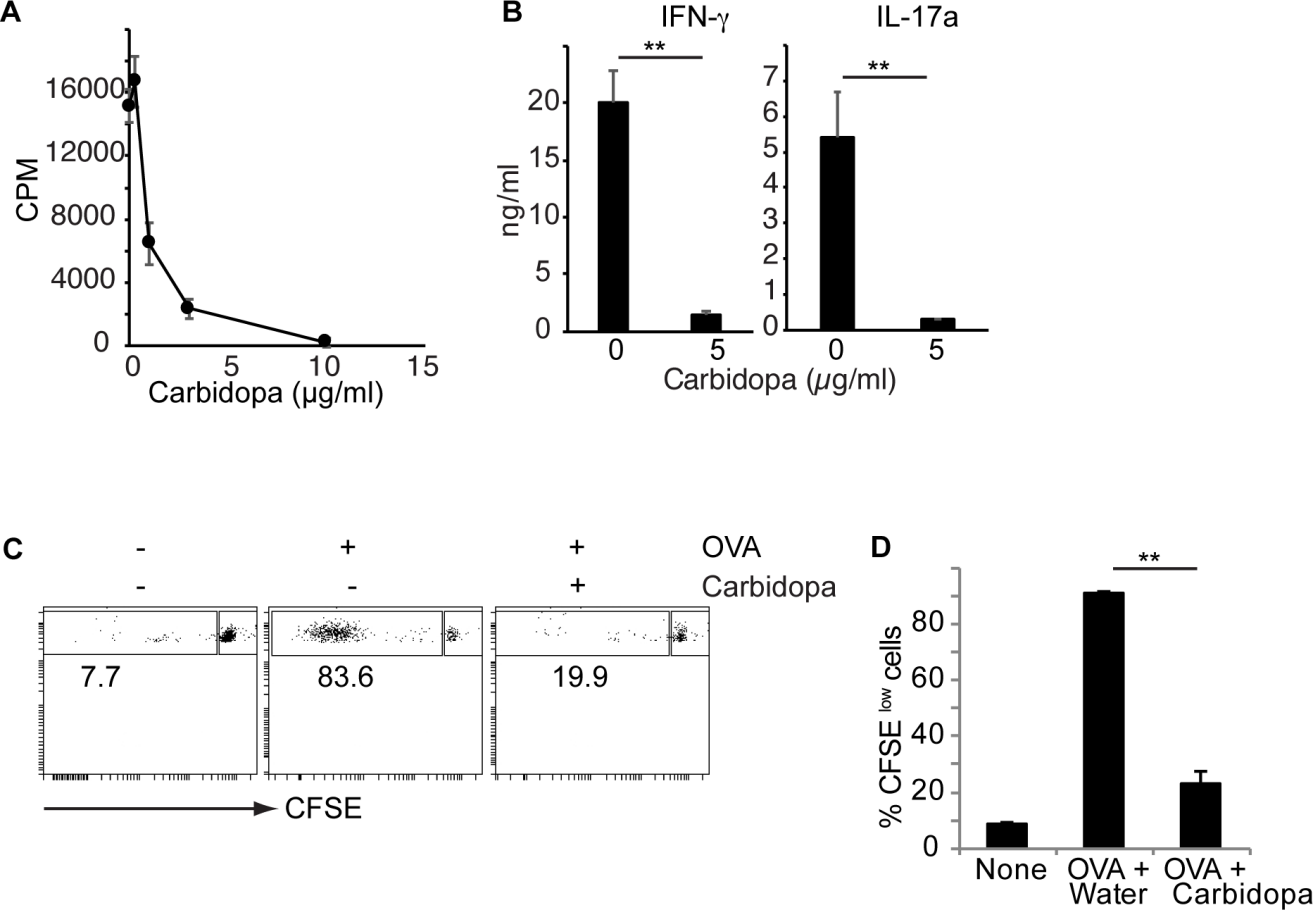
Carbidopa inhibit CD4+ T cell proliferation. **A,** CD4+ T cells were culture on anti-CD3 coated plates with or without indicated concentration of carbidopa. Two days later, proliferation of cells was determined by ^3^H-thymidine assay. Error bars represent standard deviation of triplicates. **B,** IFN-**γ** and IL-17a production by anti-CD3 activated naïve CD4+ T cells in the presence or absence of carbidopa. **C,** OTII CD4+ T cells (Thy1.1+) were transferred into WT C57BL/6 mice (Th1.2+). Next day animals were immunized with OVA. Four days later, CFSE dilution was assessed on Thy1.1+CD4+ T cells. **D,** Summary of CFSE^low^ cells from data presented in B. (n = 2–3 mice). A representative of 2 experiments is shown. ***P = <0.005, *P* value <0.05 were considered significant.

In order to understand the effect of carbidopa on T cells, proliferation of CD4+ T cells was evaluated in the presence of carbidopa. Fig 1A shows that carbidopa blocked anti-CD3 induced proliferation of CD4+ T cells in a dose dependent manner *in vitro*. In addition, Carbidopa treatment significantly inhibited production of both IFN-γ and IL-17a by anti-CD3 activated CD4+ T cells (Fig 1B). To test the effect of carbidopa *in vivo*, T cells from OTII transgenic mice on Thy1.1+ background were labeled with CFSE and transferred intravenously in to naïve B/6 mice (Thy1.2+). On the next day, all the mice were immunized with cognate ovalbumin peptide 323–339 (OVA_323-339_) emulsified in CFA. Four days later, the CFSE dilution in Thy1.1+ CD4+ T cells (OTII CD4+ T cells) in the spleens of recipients were analyzed. Only a minor fraction (8.5%) of OTII CD4+ T cells were CFSE^low^ in mice that did not receive OVA (Fig 1C and 1D). In mice that were immunized with OVA_323-339_, ~90% of the CD4+ T cells were CFSE^low^ demonstrating their activation and proliferation (Fig 1C and 1D). Addition of carbidopa in drinking water significantly decreased the fraction of OT II CD4+ T cells that were CFSE^low^ (~23%), suggesting an inhibitory effect of carbidopa on T cell proliferation *in vivo* (Fig 1C and 1D). Taken together, these data demonstrate that carbidopa inhibits T cell activation and proliferation both *in vitro* and *in vivo*.

### Carbidopa inhibits early events in T cell activation and promotes development of anti-inflammatory macrophages

**Fig 2:**
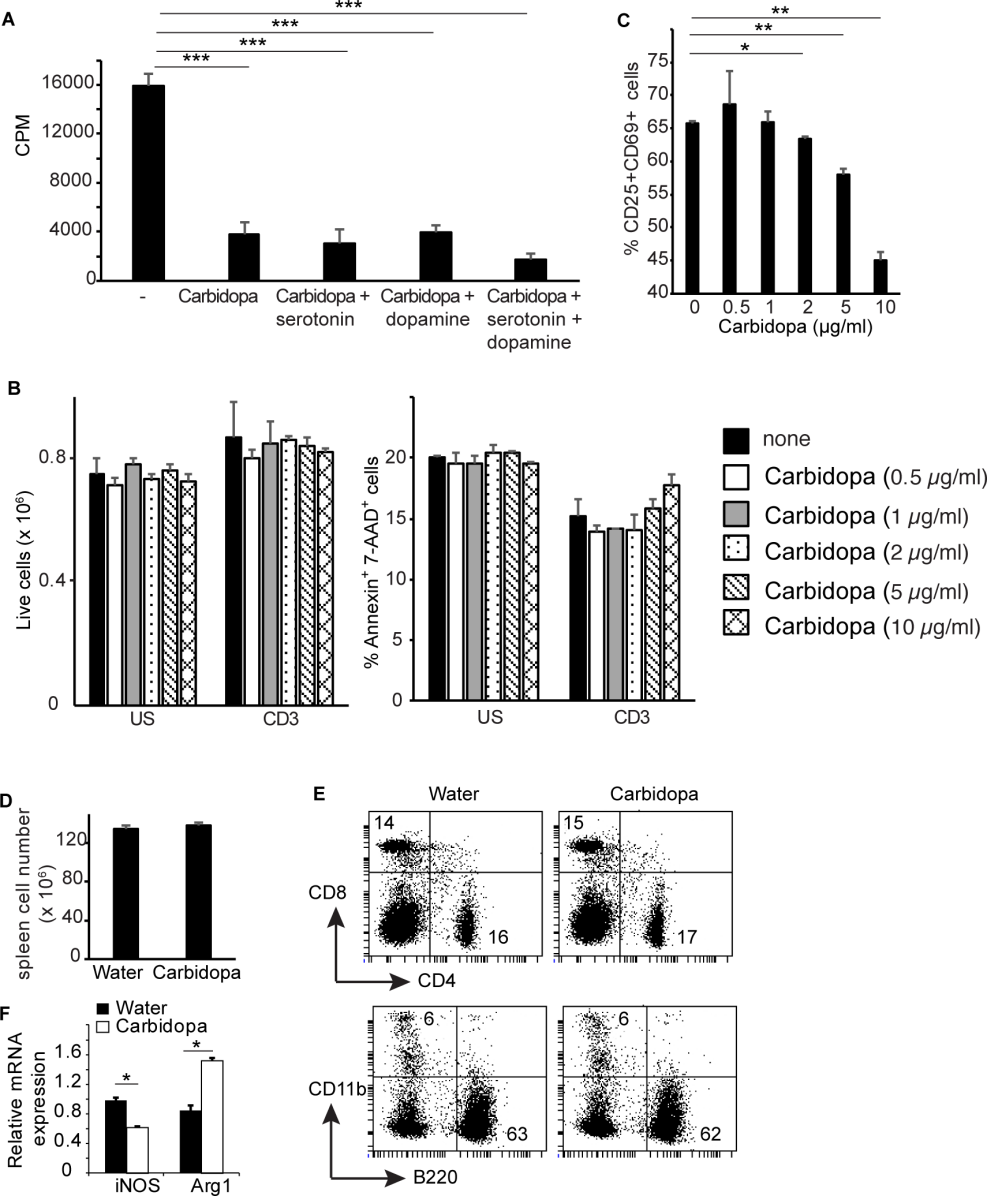
Carbidopa inhibits early events in T cell activation. **A,** T cells were activated with anti-CD3 in the presence or absence of carbidopa (5 μg/ml). Serotonin (10 μg/ml) and dopamine (10 μg/ml) were added to the indicated cultures and proliferation of T cells was determined as above. Error bars represent standard deviation triplicates. **B,** Total number of live cells (left panel) and frequency of annexin-V+ 7-AAD+ cells (right panel) recovered following overnight culture of T cells in the presence or absence of indicated concentration of carbidopa and anti-CD3. Error bar represent standard deviation of duplicates. **C,** Frequency of CD4+ T cells expressing CD25 and CD69 in response to plate bound anti-CD3 overnight activation in the presence or absence of indicated dosage of carbidopa. Unstimulated culture contained 0.2% CD25+CD69+ cells. **D,** Total number of live cells from spleen of mice treated with carbidopa as in Fig 1B (n = 4). **E**, Frequency of CD4+, CD8+, B220+ and CD11b+ cells in spleens of mice from D. The numbers represent the percent positive cells in the corresponding quadrants. **F,** Expression of iNOS and Arg1 was measured by quantitative real time PCR in splenic CD11b+ of mice from D. Error bars represent the standard deviation of mean (n = 3). *P = <0.05, **P = <0.005 and ***P = <0.0005, *P* value <0.05 were considered significant.

Carbidopa inhibits DDC, which activation leads to production of dopamine and serotonin. Both serotonin and dopamine are known to influence T cell responses. Therefore, the role of serotonin and dopamine on carbidopa-mediated inhibition of T cell responses was analyzed. T cells were stimulated with anti-CD3 in the presence or absence of carbidopa with or without serotonin or dopamine. Consistent with Fig 1, addition of carbidopa suppressed proliferation of anti-CD3 stimulated cells by ~ 75% (Fig 2A). Addition of both serotonin and dopamine alone or together to the anti-CD3 stimulated T cell cultures containing carbidopa did not change the proliferation cells (Fig 2A). Next, we tested the viability of different immune cells following carbidopa treatment. T cells were cultured overnight on anti-CD3 coated or uncoated plates in the presence or absence of graded concentration of carbidopa. Addition of carbidopa did not change the number of live cells or annexin-V+ and 7-AAD+ cells were recovered from these cultures (Fig 2B). T cell activation is a multistep process coordinated by hierarchical expression of distinct genes. One of the early events in T cell activation is the expression of CD25 and CD69. CD25 is high affinity receptor for IL-2. Binding of CD25 to IL-2 induces rapid proliferation of T cells. We tested whether carbidopa inhibits early events in T cells activation. Carbidopa blocked expression of CD25 and CD69 on CD4+ and CD8+ T cells following activation with anti-CD3 (Fig 2C and S1 Fig). Collectively, these data suggest that carbidopa inhibits early events in T cell activation. To test whether carbidopa exerts a non-specific toxic effect on immune cells *in vivo*, WT mice were treated with water containing nothing or carbidopa for 4 days as in Fig 1C. Spleens of mice treated or untreated with carbidopa possess comparable number of live cells as well as frequency of CD4+, CD8+ B220+ and CD11b+ cells (Fig 2D and 2E). Taken together, these findings indicate that carbidopa does not have toxic effects on immune cells.

Based on their ability to promote or suppress immune responses, macrophages have been classified into 2 groups, M1 (classically activated macrophages) and M2 (alternatively activated macrophages). M1 macrophages are inflammatory in nature, whereas M2 macrophages suppress inflammation. Inducible nitric oxide synthase (iNOS) and arginase 1 (Arg1) are markers for M1 and M2 macrophages respectively. We found that macrophages (CD11b+) cells from spleens of carbidopa treated animals expressed higher amounts of Arg 1 and lower levels of iNOS. This data suggest that carbidopa favors differentiation of M2 macrophages.

### Carbidopa inhibits experimental autoimmune encephalitis (EAE)

**Fig 3:**
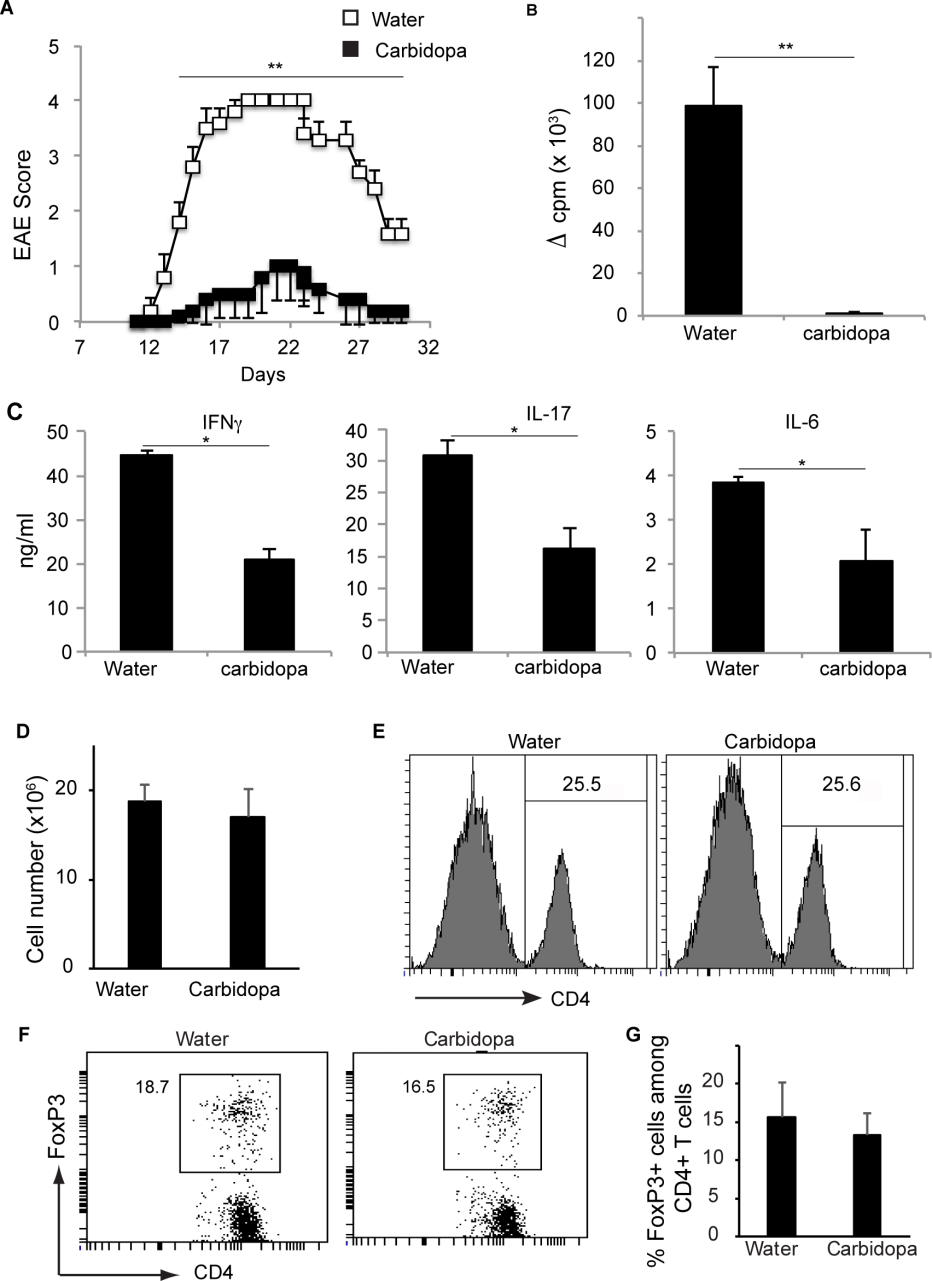
Carbidopa protects mice from EAE. **A.** Animal were given carbidopa in drinking water (filled squares) or plain water (open squares) and immunized with MOG_35-55_. Shown is the mean clinical score (n = 5). Error bars represent standard error of mean. Lymph node cells from mice immunized and treated as in A, were cultured in the presence or absence of MOG_35-55_ (20 μg/ml). Three days later, **B,** proliferation of T cells was quantified by thymidine incorporation and **C,** presence of IFN-**γ**, IL-17 and IL-6 was assessed by ELISA. Proliferation induced by no peptide was subtracted from that induced by MOG_35-55_ (20 μg/ml) to obtain ΔCPM. Error bars represent standard deviation of mean (n = 5). **D,** Number of live cells and **E,** frequency of CD4+ T cells in inguinal lymph nodes from mice treated as in A. **F,** FoxP3 expression by CD4+ T cells from E. **G,** summary of FoxP3 by CD4+ T cells from F. Error bars represent standard deviation of mean (n = 4). *P = <0.05, and **P = <0.005, *P* value <0.05 were considered significant.

Carbidopa is used for management of Parkinson’s disease. Several evidences indicate inflammation as one of the factors responsible for the induction and progression of PD. Carbidopa does not cross the blood-brain barrier. However, priming of T cells against self-antigens most likely happens in lymph nodes, which are located outside of the brain. Therefore, we tested whether carbidopa can inhibit T cell mediated autoimmunity in the brain. C57BL/6 mice were immunized with myelin oligodendrocyte glycoprotein peptide 35–55 (MOG_35-55_) and treated with plain water (control) or water containing carbidopa. Mice receiving plain water started showing signs of limb paralysis at ~ 2 weeks after immunization (Fig 3). Between days 19–23 after immunization the disease was most severe (mean clinical score 4) in this group followed by a steady decline in the gravity of the disease. In contrast, the mice receiving carbidopa in their drinking water showed drastically reduced severity which was ~ 4 fold lower at peak than the group fed with plain drinking water (Fig 3A).

**Fig 4:**
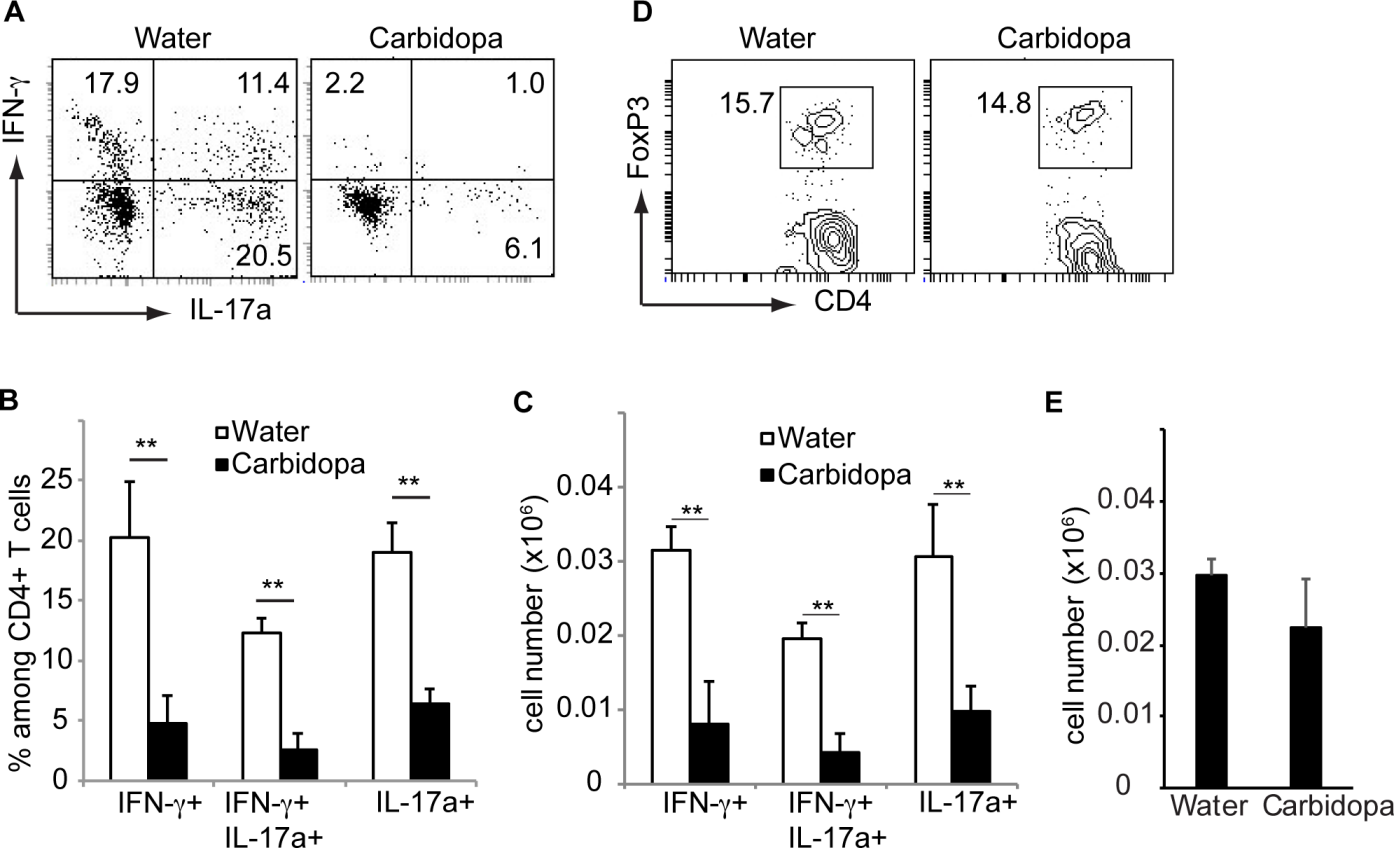
Carbidopa decreases number of pathogenic CD4+ T cells during EAE. Immune cells were isolated from brain of mice as immunized in Fig 2 were stimulated with PMA + ionomycin for 5 hours stained with indicated cytokines and CD4. **A,** Shown is IFN-**γ** and IL-17 expression by CD4+ T cells. **B,** Summary of data shown in A. **C,** Quantification of number of CD4+ T cells producing indicated cytokines. **D,** Frequency and **E,** number of Treg cells in the brain of mice as immunized in Fig 2. Error bars represent standard deviation of mean (n = 4). **P = <0.005, *P* value <0.05 were considered significant.

Next, lymph node cells from mice immunized with MOG_35-55_ were cultured in the presence or absence of cognate peptide, and proliferation of cells and cytokine production in the culture supernatant was measured. Lymph nodes cells from mice immunized with MOG_35-55_ proliferated more vigorously in response to *ex vivo* challenge with MOG_35-55_ than those from mice immunized with MOG_35-55_ and treated with carbidopa (Fig 3B). Accordingly, MOG_35-55_ induced significantly reduced amounts of IFN-γ, IL-17 and IL-6 in lymph node cells from carbidopa treated mice than untreated mice (Fig 3C). Reduced proliferation of lymph node cells from carbidopa treated mice was not due to the presence of a decreased number of CD4+ T cells or an increased number of Treg cells, because lymph nodes from carbidopa treated mice contain similar number of total live cells, and a similar frequency of CD4+ T cells and Treg cells (CD4+Foxp3+) (Fig 4D–4G).

To evaluate the cytokine production by CD4+ T cells infiltrating the central nervous system, lymphocytes from brain were stimulated with phorbol 12-myristate 13-acetate (PMA) + ionomycin and stained with antibodies against IFN-γ and IL-17. CD4+ T cells producing either IFN-γ, 17, or both IFN-γ and IL-17 were present in these cultures. Carbidopa treatment significantly reduced proportions and number of CD4+ T cells producing IFN-γ in brains of mice (Fig 4A–4C). Frequency and number of central nervous system (CNS) infiltrating CD4+ T cells producing IL-17a was also significantly inhibited following carbidopa treatment (Fig 4A–4C). Similarly, CD4+ T cells producing both IFN-γ and IL-17 was reduced by ~5 fold in brains of carbidopa treated animals (Fig 4A–4C). In contrast, carbidopa treatment did not change the frequency and number of Treg in brains of mice (Fig 4D and 4E). Taken together, these data demonstrate that carbidopa inhibit MOG_35-55_ driven EAE in animal model.

### Blockade of collagen induced autoimmune arthritis (CIA) by carbidopa

**Fig 5:**
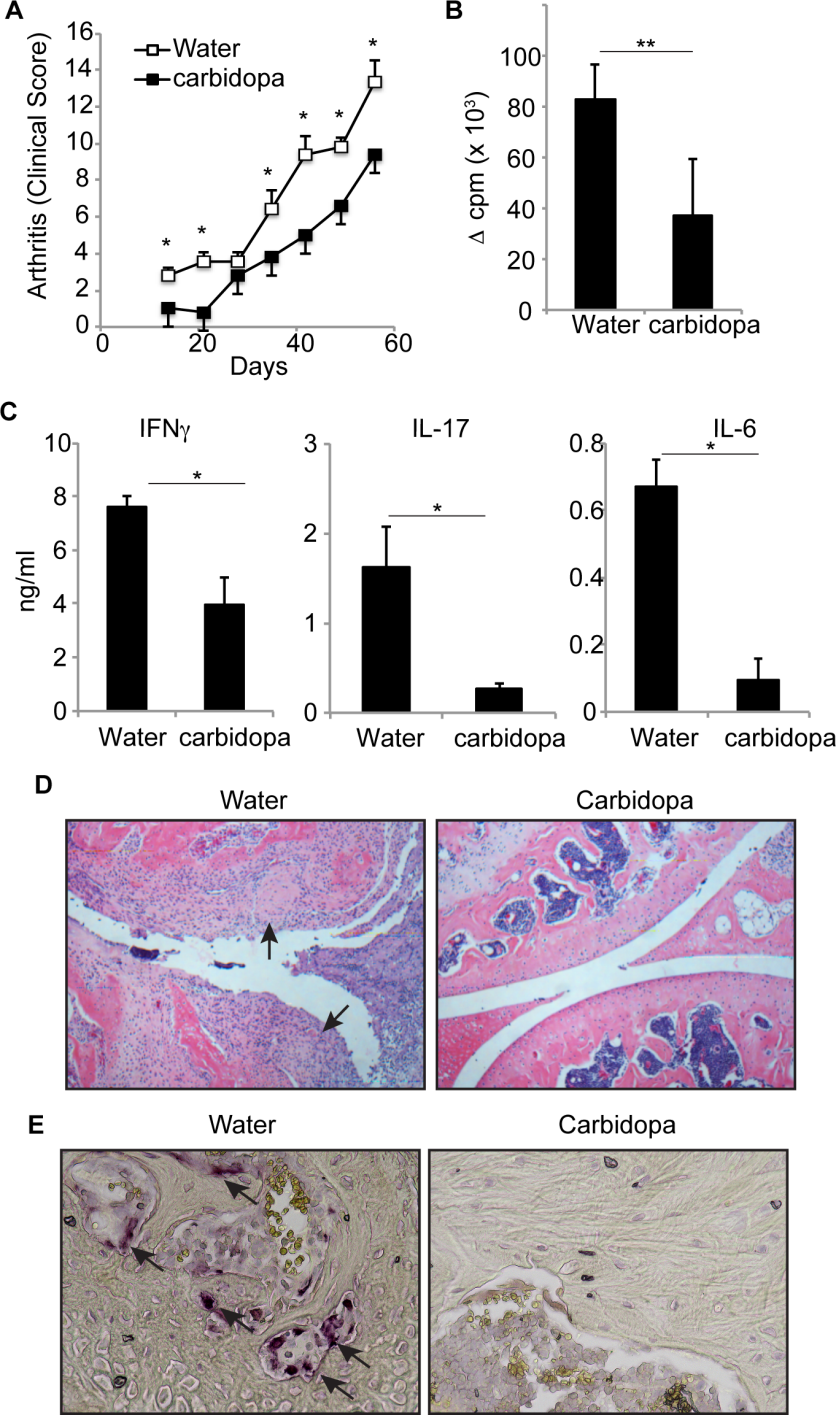
Carbidopa treatment protects joints from collagen induced autoimmune arthritis. Bovine type II collagen immunized mice were either given drinking water containing carbidopa or plain water. **A.** Clinical score for arthritis development. **B and C**. Lymph node cells from mice immunized as in A were challenged in vitro with collagen (100μg/ml) and proliferation and cytokine production was analyzed as in Fig 2. Error bars represent standard deviation of mean (n = 5). **D.** A photograph of Hematoxylin & Eosin staining of the articular knee joints of collagen induced autoimmune mouse supplemented with water only or drug Carbidopa in water. The arrows indicate loss of cartilage in knee of mice supplemented with water whereas Carbidopa prevent this effect (original magnification 200X). **E.** A photograph of TRAP staining of the bones of the mice treated as in A. The arrows indicate the presence of osteoclasts. (original magnification 400X). *P = <0.05, and **P = <0.005, *P* value <0.05 were considered significant.

We also tested whether carbidopa could inhibit T cell driven pathology outside of the central nervous system. For this purpose, we used a well-established model of collagen induced arthritis in DBA/1 mice. Mice were immunized with bovine type II collagen emulsified in CFA. Half of the mice were given carbidopa in drinking water, and other half received plain drinking water. Mice were monitored for swelling, erythema, digit deformity and ankylosis of paws. Mice started showing signs of arthritis ~ 2 weeks after immunization and severity of disease progressively increased until mice were observed, 2 months after immunization. Severity of the disease was significantly lower in the group receiving carbidopa in drinking water than group fed with plain water (Fig 5A). In accordance, carbidopa treatment decreased joint inflammation and cartilage destruction (Fig 5D).

To test whether diminished T cell priming is the reason for reduced development of arthritis in carbidopa treated group, lymph node cells from immunized mice were cultured in the presence of collagen and proliferation of cells and production of cytokine was quantified. Fig 5B shows that lymph node cells from carbidopa treated group exhibited significantly reduced collagen induced proliferation than from control mice. Similarly, collagen induced production of IFN-γ, IL-17, and IL-6 was greatly diminished in animals that received carbidopa drinking water than mice fed with plain water (Fig 5C). Osteoclasts are multinucleated cells that cause bone resorption during arthritis. Therefore, presence of osteoclast in the bones was evaluated by tartrate-resistant acidic phosphatase (TRAP) staining. Osteoclast were abundantly present in bones of mice that received plain water. Consistent with decreased arthritis clinical score and decreased joint inflammation, carbidopa treatment also inhibited presence of osteoclasts in bones (Fig 5E). Taken together, these data demonstrate that carbidopa inhibits collagen induced T cells priming and arthritis.

## Discussions

Data presented here demonstrate that carbidopa, which is being used for management of PD, inhibits T cell activation *in vitro*, *in vivo*, and T cell mediated autoimmunity [11, 21–25]. DDC, which catalyzes production of serotonin and dopamine is a known target of carbidopa. Failure of serotonin and dopamine to overcome the inhibitory effect of carbidopa on T cells suggests presence of novel carbidopa target which plays a key role in T cell activation. In this study, we report for the first time to our knowledge that carbidopa, a specific inhibitor of DDC, inhibits T cell responses and autoimmunity. Carbidopa has been used by human for many years without significant side effects, and our findings suggest potential therapeutic uses of carbidopa for management and/or treatment inflammatory and autoimmune disorders in humans.

We show that carbidopa favors polarization of macrophages towards anti-inflammatory M2 phenotype. Certain cytokines such as IFN-γ and GM-CSF promote the inflammatory M1 macrophages [26, 27]. Although in general M1 cytokines promote immune pathology, GM-CSF have been shown to either promote or suppress inflammations in a context dependent manner. GM-CSF worsens the outcomes in EAE and arthritis, whereas it improves the inflammation in Crohn’s disease, Type-1 diabetes, and Mysthenia gravis [26–29]. Available data suggest that pro-inflammatory and anti-inflammatory effect of GM-CSF may depend on other cytokines and factors present in microenvironment and thus it will be interesting to test whether carbidopa may affect the immunomodulatory effects of GM-CSF. Antigen presenting cells such as CD103+ dendritic cells or macrophages present in intestine or anterior chamber of eye, respectively display anti-inflammatory properties [30, 31]. Introduction of antigens to anterior chamber of eye suppresses delayed-type hypersensitivity (DTH) responses against MOG, myelin basic protein (MBP) and type II collagen [31–34]. Macrophages present in anterior chamber of eye migrate to spleen and induce tolerance via induction of regulatory T cells. It is highly possible that carbidopa-mediated inhibition of iNOS and promotion of Arg1 in splenic macrophages or those migrating from anterior chamber of eye to spleen may enhance their tolerogenic potential in an additive or synergistic way. Future studies are warranted to test these possibilities.

There are several evidence of involvement of T cell mediated inflammation in promoting pathology of PD [35]. IL-17 production by T cells exacerbates Methyl-phenyl-tetrahydropyridine (MPTP) induced PD in animal models [36]. PD individuals have increased number of activated and memory T cells in blood, which positively correlates with motor dysfunction [37, 38]. At present, it is unclear whether this increase in activated T cells in peripheral blood is cause or consequence of PD. If activated/memory T cells from blood migrate to CNS to cause pathology, suppression of these T cells by carbidopa may be benefit PD individuals. Our finding that carbidopa potently inhibits T cell responses and T cell mediated autoimmunity in two different animal models, demonstrates a strong evidence of immunosuppressive activity of this compound. Our data indicate that PD individuals consuming carbidopa may show immune suppression and thus will be at enhanced risk of infections. In light of our findings, a detailed mechanistic study to illustrate molecular mechanisms targeted by carbidopa leading to inhibition of T cell responses is warranted to understand immunosuppressive role of this compound and its use in prevention and/or treatment of inflammatory and autoimmune disorders.

## Supporting information

S1 Fig Carbidopa inhibits CD25 and Cd69 expression in anti-CD3 activated CD8+ T cells. CD25 and CD69 expression by CD8+ T cells in response to overnight anti-CD3 stimulation in the presence or absence of indicated dosage of carbidopa. Unstimulated culture contained 0.05% CD25+CD69+ cells.
(TIF)
