# Supplementary figures and images for: Carbidopa, a drug in use for management of Parkinson disease inhibits T cell activation and autoimmunity
Source: PLoS One. 2017 Sep 12;12(9):e0183484. doi: 10.1371/journal.pone.0183484 (PMC5595290; doi:10.1371/journal.pone.0183484)

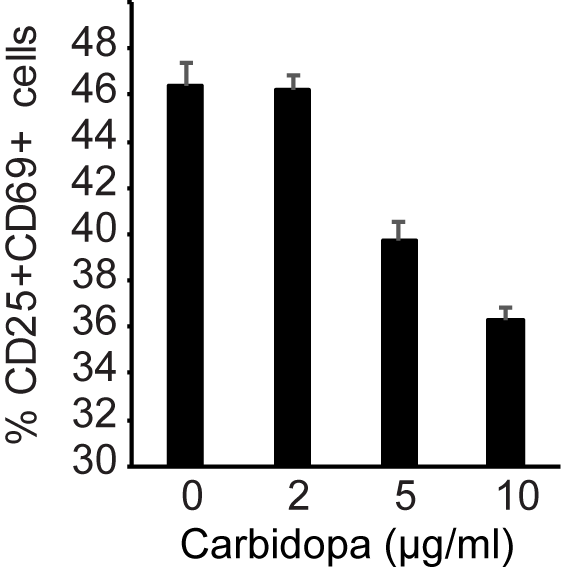

Supplement: S1 Fig — CD25 and CD69 expression by CD8+ T cells in response to overnight anti-CD3 stimulation in the presence or absence of indicated dosage of carbidopa. Unstimulated culture contained 0.05% CD25+CD69+ cells. (TIF) [file pone.0183484.s001.tif]
